# Fine-Needle Pricking Test of the Parathyroid Gland during Thyroid Surgery in Predicting Parathyroid Function

**DOI:** 10.1155/2022/8747680

**Published:** 2022-06-25

**Authors:** Ying-Jun Wu, Jian-Biao Wang, Fei-Bo Li, Lei Jin, Liang Zhou, Lei Xie

**Affiliations:** ^1^Department of Operation Room Nursing, The Affiliated Sir Run Run Shaw Hospital, School of Medicine, Zhejiang University, Hangzhou, Zhejiang 310016, China; ^2^Department of Head and Neck Surgery, The Affiliated Sir Run Run Shaw Hospital, School of Medicine, Zhejiang University, Hangzhou, Zhejiang 310016, China; ^3^Second Department of General Surgery, Zhejiang Putuo Hospital, Zhoushan, Zhejiang 316100, China

## Abstract

**Background:**

Permanent hypoparathyroidism is a serious complication following total thyroidectomy plus central neck dissection (CND). How to evaluate the vascularization of the parathyroid gland in real time is a major concern of thyroid surgeons. This study aimed to evaluate the fine-needle pricking (FNP) test in predicting parathyroid gland function.

**Methods:**

The FNP test was performed in patients undergoing total thyroidectomy plus CND between January 1, 2014, and December 31, 2019, to visualize the vascularization of the parathyroid glands. Patients were classified according to the number of parathyroid glands preserved *in situ* with excellent vascularity (PGPIEV) demonstrated by FNP: group 0 (without PGPIEV), group 1 (with one PGPIEV), group 2 (with two PGPIEV), group 3 (with three PGPIEV), and group 4 (with four PGPIEV).

**Results:**

A total of 608 patients with four parathyroid glands underwent FNP testing during thyroidectomy. At least one PGPIEV was demonstrated by FNP testing in 581 patients who had intact parathyroid hormone (iPTH) levels in the normal range after the operation. The prevalence of hypocalcemia decreased from 77.8% in group 0 to 9.8% in group 4 (*P* < 0.001), and the incidence of hypoparathyroidism decreased from 44.4% in group 0 to 0% in groups 1–4 (*P* < 0.001). iPTH concentrations on postoperative day 1 were positively correlated with PGPIEV groups (increased from 14.58 ng/l in group 0 to 45.22 ng/l in group 4, *P* < 0.001).

**Conclusions:**

The FNP test is a safe and reliable method to predict parathyroid function. One PGPIEV demonstrated by the FNP test rules out the possibility of patients developing hypoparathyroidism.

## 1. Introduction

Permanent hypoparathyroidism is the most common serious complication after total thyroidectomy, with an estimated incidence of 4–11% [1–8]. Patients with permanent hypoparathyroidism not only need a daily intake of calcium/vitamin D supplements but also have an increased risk of renal insufficiency, malignancy [7], and death [8].

The main causes of hypoparathyroidism after total thyroidectomy are intraoperative damage to the parathyroid glands by trauma, inadvertent parathyroid gland removal, or devascularization. The extent of damage to the parathyroid glands is difficult to predict during surgery. Some studies proposed that half of one normal parathyroid gland can produce sufficient parathyroid hormone (PTH) [9, 10]. In order to avoid permanent hypoparathyroidism, parathyroid autotransplantation can be performed to salvage the devascularized parathyroid gland [11–16]. Typically, the strategic decision on how to select one parathyroid gland for autotransplantation relies on the viability of the gland. “Dead” parathyroid (without blood supply) has much greater odds of resurrection after autotransplantation than that remaining *in situ* [17]. The fine-needle pricking (FNP) test is a simple tool for evaluating the blood supply of the parathyroid gland. However, no studies have yet assessed the reliability and safety of the FNP test.

Thus, the present study aimed to evaluate the use of the FNP test in predicting parathyroid gland function, the method to identify whether one parathyroid gland should be autotransplanted or remain *in situ*, and the absence of postoperative hypoparathyroidism in patients in whom the test could demonstrate good vascularization of at least one parathyroid gland.

## 2. Materials and Methods

### 2.1. Patients

A retrospective review of patients with thyroid cancer who underwent total thyroidectomy plus ipsilateral or bilateral central neck dissection (CND) between January 1, 2014, and December 31, 2019, was conducted. The exclusion criteria were previous thyroidectomy, preoperative hypo- or hyperparathyroidism, and osteoporosis requiring calcium or vitamin D treatment.

The study protocol was approved by the Ethics Committee of the Affiliated Sir Run Run Shaw Hospital, Zhejiang University School of Medicine.

### 2.2. Surgical Procedure and FNP Test Protocol

Thyroid surgery was performed by two surgeons or two surgical fellows under their direct supervision. All patients underwent direct laryngoscopy preoperatively for the evaluation of vocal cord mobility. An intraoperative neuromonitoring instrument (NIM-Response 3.0 System; Medtronic Xomed, Jacksonville, FL, USA) was used for all patients. After the operation, laryngoscopy was performed only in patients with postoperative hoarseness. The extent of CND was assessed according to the American Thyroid Association guidelines [18]. Bilateral CND involved the removal of the laryngeal, pretracheal, and both the right and left paratracheal nodal basins. Unilateral CND involved the removal of the prelaryngeal, pretracheal, and one paratracheal nodal basin.

Thyroid lobectomy was performed according to the “meticulous capsular dissection” concept [19]. The parathyroid glands were not routinely and deliberately exposed during thyroidectomy. In case a parathyroid gland was encountered during thyroid lobectomy, best efforts were made to preserve the gland *in situ* with its feeding vessel. CND was performed according to the “layer of thymus-blood vessel-inferior parathyroid gland” concept, as described previously [20]. However, best efforts were made during the CND to search for and preserve the inferior parathyroid gland.

**Figure 1:**
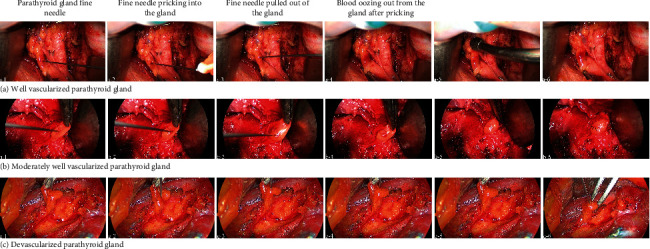
Representative parathyroid gland fine-needle pricking (FNP) test images. (a) A well-vascularized parathyroid gland (FNP score of 2). a-5, the blood covering the gland was cleared by suction. At a-6, blood was still oozing out from the incision of the gland. (b) A moderately well-vascularized parathyroid gland (FNP of score 1). b-5, the blood covering the gland was wiped by gauze. b-6, small and slow blood oozing from the incision of the gland was detected (black arrow). (c) A devascularized parathyroid gland (FNP score of 0). c-5, no blood oozing out from the incision of the gland waited for 10 to 20 seconds after the pricking. c-6, no blood oozing out after more times pricking of the gland.

After thyroid resection and CND, all identified parathyroid glands were scored for viability from grade 0 (no vascularity) to 2 (excellent vascularity) using the FNP test. The preserved parathyroid gland was handled with forceps; then, the parenchyma was pricked with a 25G injection needle. The bleeding from the small hole in the gland was assessed. A bleeding score for the FNP was established as follows: FNP 0, the parathyroid has no blood oozing after the pricking, indicating the gland is not vascularized; FNP 2, the parathyroid has blood effusing out after pricking, and the blood oozing persists when the blood covering the gland is cleared by suction, indicating that the gland is well vascularized; FNP 1, the parathyroid is oozing blood but slowly and in small volume, which persisted even after wiping with a gauze, suggesting that the gland is partially vascularized (Figure 1).

Parathyroid glands with an FNP score of 1 or 2 were retained *in situ*. If the FNP score was 0, the gland was routinely autotransplanted into the sternocleidomastoid muscle.

The parathyroid gland preserved *in situ* with excellent vascularity (PGPIEV) score was calculated according to the number of parathyroid glands preserved *in situ* with FNP score 2. Subsequently, the patients were classified according to the PGPIEV number as group 0 (zero PGPIEV), group 1 (one PGPIEV), group 2 (two PGPIEV), group 3 (three PGPIEV), and group 4 (four PGPIEV).

The standard postoperative protocol of the institution was followed for all patients. Patients were not routinely administered calcium after total thyroidectomy plus CND unless the patient developed symptomatic hypocalcemia. Calcium supplements containing 750 mg of calcium carbonate plus 60 units of vitamin D3 were administered orally twice daily to treat symptomatic hypocalcemia. A calcium gluconate injection was prescribed for persistent symptomatic hypocalcemia after oral calcium treatment.

### 2.3. Laboratory Assays

Serum calcium and intact parathyroid hormone (iPTH) levels were assessed in patients before the surgery and every morning (06.00 a.m.) thereafter until discharge. Most patients were discharged on postoperative day (POD) 3, depending on the volume of drainage fluid. Serum iPTH levels were measured on a Roche Cobas E601 instrument (Hitachi High-Technologies, Tokyo, Japan) (normal range 15–65 ng/l). The serum calcium levels were measured on an Abbott Aeroset Automated Instrument Analyzer (Toshiba Medical Systems, Tochigi-ken, Japan) (normal range 2.11–2.52 mmol/l). Ionized calcium amounts were not determined separately in this study.

### 2.4. Definition of Hypoparathyroidism

Transient hypoparathyroidism was defined as an iPTH level below the normal range (15 ng/l) after the surgery [21–23]. Hypocalcemia was defined as the serum calcium level <2 mmol/l (8 mg/dl) or the requirement for calcium supplementation to treat the clinical symptoms of hypocalcemia, such as distal digital paresthesia or tetany, during the hospital stay [10, 24]. Permanent hypoparathyroidism was defined as subnormal serum iPTH levels, calcium levels <2 mmol/l, or a requirement for calcium and/or vitamin D supplements to treat hypocalcemia-related symptoms for >6 months.

### 2.5. Statistical Analysis

Continuous data were presented as mean ± standard deviation. The normal distribution of quantitative variables was assessed using the Kolmogorov–Smirnov test. The comparison of proportions was investigated using the Pearson's *χ*^2^ test. A one-way analysis of variance (ANOVA) or the Kruskal–Wallis test was used for quantitative variables. *P* < 0.050 indicated a statistically significant difference. The data were analyzed using SPSS version 16.0 (IBM, NY, USA).

## 3. Results

### 3.1. Patient Characteristics

**Figure 2:**
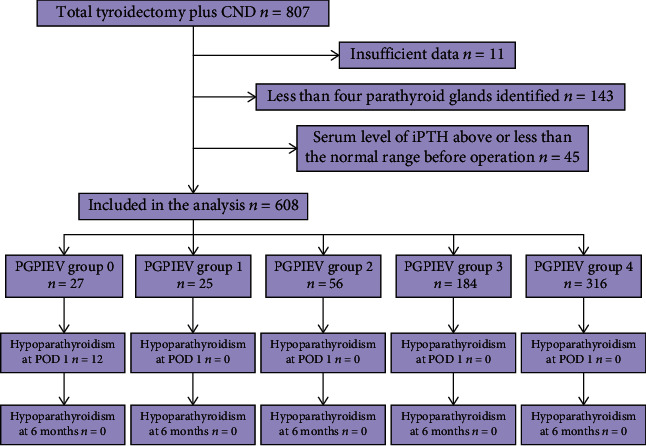
Flowchart for the study of parathyroid gland fine-needle pricking tests to predict parathyroid function. The PGPIEV group indicates the number of *in situ* preserved parathyroid glands with excellent vascularity (see text for details). Hypoparathyroidism was defined by an intact parathyroid hormone level below 15 ng/l. CND, central neck dissection; POD, postoperative day.

A total of 807 consecutive patients underwent total thyroidectomy plus CND in the clinical treatment group of Dr. Xie at the Department of Head and Neck Surgery between January 1, 2014, and December 31, 2019. A flowchart of the study is illustrated in Figure 2. Of these, 608 patients whose all four parathyroid glands were identified during the operation were included in the analysis.  Table 1 provides the clinical data of the 608 patients who underwent total thyroidectomy plus CND, followed by the parathyroid gland FNP test.

### 3.2. FNP Test Results for Parathyroid Glands

**Figure 3:**
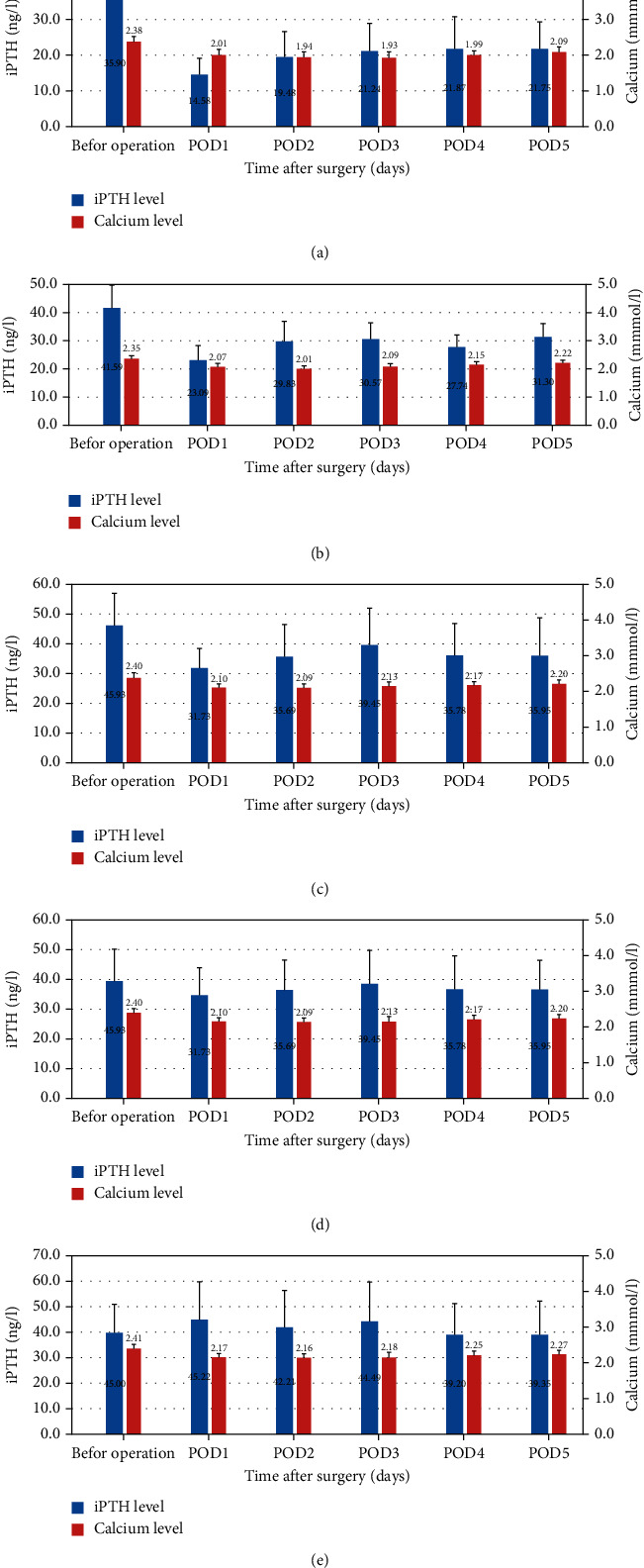
Mean serum levels of intact parathyroid hormone (iPTH) and calcium during the perioperative period in different parathyroid glands preserved in situ with excellent vascularity (PGPIEV) groups. (a), PGPIEV group 0; (b), PGPIEV group 1; (c), PGPIEV group 2; (d), PGPIEV group 3; (e), PGPIEV group 4. POD, postoperative day.

The results of the FNP test for parathyroid glands are presented in Table 2. Of the 608 patients who underwent FNP testing, 581 had an FNP score of 2 for at least one parathyroid gland. The PGPIEV group 0 included 27 patients, of whom 12 developed transient hypoparathyroidisms (Figure 3(a), Figure S1a supplementary information). The postoperative iPTH levels were in the normal range in 25 patients enrolled in the PGPIEV group 1 (Figure 3(b), Figure S1b supplementary information). Additionally, the postoperative iPTH levels were in the normal range in patients included in PGPIEV groups 2–4 (56 in group 2, 184 in group 3, and 316 in group 4) (Figures 3(c)–3(e), Figures S1c–S1e supplementary information). Therefore, in the 581 patients with at least one parathyroid gland with a FNP score of 2, postoperative iPTH levels were in the normal range.

The serum iPTH concentration at POD 1 and its proportion of the initial value (before operation) are the indicators of the function of the *in situ* preserved parathyroid glands. The patients in PGPIEV group 0 had the lowest POD 1 iPTH levels (14.58 ng/l), while patients in PGPIEV group 4 had the highest POD 1 iPTH levels (45.22 ng/l) (Figure 3, Table S1 supplementary information). The iPTH levels on POD 1 were positively correlated with the PGPIEV score (*P* < 0.001) (Table 3). The proportion of iPTH levels on POD 1 of the initial value (before operation) increased significantly from 42.7% in the PGPIEV group 0 to 114.9% in group 4 (*P* < 0.001) (Table 3). Therefore, the PGPIEV score was positively correlated with the function of *in situ* preserved parathyroid glands.

21/27 (77.8%) patients developed hypocalcemia in the PGPIEV group 0. Asymptomatic hypocalcemia was detected in 15 (60.0%) patients in PGPIEV group 1, in 15 (26.8%) patients in PGPIEV group 2, in 18 (9.8%) patients in PGPIEV group 3, and in 31 (9.8%) patients in PGPIEV group 4 (Table 3). Transient hypoparathyroidism was detected in 12 (44.4%) patients in PGPIEV group 0. None of the patients in PGPIEV groups 1–4 developed hypoparathyroidism (Table 3). A low PGPIEV score was correlated with a high prevalence of transient hypocalcemia (*P* < 0.001) and hypoparathyroidism (*P* < 0.001) (Table 3).

### 3.3. Adverse Reactions and Follow-Up

The FNP test did not give rise to any adverse reactions. The active bleeding by incision of the parathyroid gland parenchyma after the FNP test was stopped without needing hemostasis to control the bleeding. After > 6 months of follow-up, none of the 608 patients developed permanent hypoparathyroidism.

## 4. Discussion

The present study has shown that the FNP test of the parathyroid gland in patients undergoing total thyroidectomy plus CND is safe, and the results suggest an excellent correlation between FNP score and parathyroid function. The postoperative iPTH levels were normal in all patients who had at least one well-vascularized parathyroid gland, according to the FNP test. Thus, the FNP test is a reliable tool to decide whether one parathyroid gland should be autotransplanted or left *in situ*.

Currently, postoperative hypoparathyroidism is the most common complication following total thyroidectomy plus CND [25]. Especially, permanent hypoparathyroidism increases the risk of malignancy [7] and death [8], leading to cerebral, vascular, ocular, and renal damage and a significant decline in the quality of life [26, 27]. Parathyroid identification and preservation *in situ* with an optimal vascular supply is the mainstay of safe thyroid surgery. However, some studies reported that parathyroid autotransplantation is an effective method to reduce the risk of permanent postoperative hypoparathyroidism, but is associated with a markedly high prevalence of transient hypoparathyroidism [11–16, 28]. Therefore, a reliable tool that can help the surgeon decide about the autotransplantation of one parathyroid gland during thyroidectomy is essential.

Most thyroid surgeons rely on visual inspection of a parathyroid gland to decide whether it is well-vascularized or whether it should be autotransplanted. However, observation of the parathyroid color change is not reliable [29]. High rates (4.1–16.2%) of permanent hypoparathyroidism have been reported after total thyroidectomy and CND in some thyroid centers [4–6, 30].

The FNP test enables a direct evaluation of the blood supply to the parathyroid gland and assists in selecting patients who require parathyroid autotransplantation when a nonvascularized parathyroid gland is identified. In this study, parathyroid glands with an FNP score of 0 were routinely autotransplanted into the sternocleidomastoid muscle, and those with an FNP score of 1 or 2 were retained *in situ*. A total of 163 parathyroid glands were scored 0. Of the 163 parathyroid glands, 23 were removed with the tumors due to tumor infiltration, 17 could not be preserved *in situ*, and 123 were autotransplanted based on the FNP test results indicating the absence of blood supply. According to this protocol, 12 of 608 (2.0%) patients exhibited transient hypoparathyroidism, but none of the 608 patients developed permanent hypoparathyroidism.

An early and accurate predictor of hypoparathyroidism is critical as it improves the selection of patients for early discharge and decreases the rate of readmission. The estimation of the iPTH level at different time points after thyroidectomy is the most commonly used method to evaluate parathyroid function [23, 31]. However, the positive predictive value of the absence of hypoparathyroidism reported by other authors was no prospects [32, 33]. Moreover, considering the cost, the assay to estimate the serum iPTH level was not available anytime in most hospitals; also, it was time-consuming. A rapid intraoperative parathyroid hormone assay at different time points may be used for clinical research but is difficult for routine clinical practice.

Parathyroid gland angiography with indocyanine green fluorescence has been reported as a reliable method to predict parathyroid function [21, 22]. Nonetheless, it needs an expensive laparoscopic imaging camera system, which is not routinely available in most hospitals, and administering intravenous indocyanine green is a contraindication for iodine allergy patients. The FNP test is a safe, simple, and reliable method to predict parathyroid function during thyroid surgery in real time. In this study, we only included patients with all four parathyroid glands identified during thyroid surgery. Strikingly, 25 patients had only one well-vascularized parathyroid gland as demonstrated by the FNP test. The postoperative iPTH levels in these patients were in the normal range. Together, these findings suggest that having at least one FNP score of 2 for the parathyroid gland rules out the possibility of patients developing hypoparathyroidism and symptomatic hypocalcemia.

The shortcoming of the FNP test is the identification of parathyroid glands with high accuracy. Even the most experienced thyroid surgeons can misinterpret other anatomical structures, such as thyroid and thymus nodules or lymph nodes, as a parathyroid gland. If good vascularization of nonparathyroid tissue was demonstrated by the FNP test, it would lead to a false reassurance that the patients would not develop hypoparathyroidism. The immunochromatographic test strip to detect fine-needle aspirated tissue PTH using the immune colloidal gold technique for rapid intraoperative parathyroid identification [34] maybe a promising method to assist surgeons in identifying parathyroid glands with certainty.

In conclusion, this study demonstrated that when at least one parathyroid gland was well-vascularized after total thyroidectomy plus CND, the postoperative iPTH levels were within the normal range in all patients. The PGPIEV score shows an excellent correlation with postoperative parathyroid function. The FNP test could be a safe, simple, and reliable tool for assessing the function of parathyroid glands, following thyroid resection in real time and deciding whether one parathyroid gland should be autotransplanted or retained *in situ* during thyroid surgery.

## Figures and Tables

**Table 1:** Demographic and clinical details of patients who underwent total thyroidectomy plus central neck dissection.

	No. of patients (*n* = 608)
Age (years)^*∗*^	43.0 (12.3)
Sex ratio (M : F)	162 : 446
Tumor size on histology (cm)†	0.90 (0.20–6.00)
Overall lymph node yield in CND†	
Retrieved	13.0 (2.0–58.0)
Metastatic	1.0 (0.0–47.0)
Central neck lymph node metastases (*n* (%))	375 (61.7%)
Indication for surgery	
Papillary thyroid carcinoma	599
Medullary thyroid carcinoma	6
Follicular thyroid carcinoma	2
Poorly differentiated thyroid carcinoma	1
Extent of surgery	
Total thyroidectomy + ipsilateral central neck dissection	184
Total thyroidecomy + bilateral central neck dissection	252
Total thyroidectomy + ipsilateral central and lateral neck dissection	12
Total thyroidectomy + bilateral central + ipsilateral lateral neck dissection	138
Total thyroidectomy + bilateral central and lateral neck dissection	22

Values are ^*∗*^ mean (standard deviation) and ^†^median (range). CND, central neck dissection.

**Table 2:** Fine-needle pricking test classification of parathyroid glands in patients who underwent total thyroidectomy plus central neck dissection.

	Right superior parathyroid	Right inferior parathyroid	Left superior parathyroid	Left inferior Parathyroid
Total no. of parathyroid glands identified	608	608	608	608
Score 2	447	518	456	532
Score 1	137	29	126	24
Score 0	24	61	26	52

Fine-needle pricking (FNP) score: FNP 0, the parathyroid has no blood oozing after the pricking, indicating the gland is not vascularized; FNP 2, the parathyroid has blood effusing out after the pricking, indicating that the gland is well vascularized; FNP 1, the parathyroid has blood oozing, but it is slow and small, and the slow blood oozing persist when it was wiped by gauze, suggesting that the gland is partially vascularized.

**Table 3:** Influence of parathyroid glands preserved *in situ* with excellent vascularity on the risk of hypocalcemia and hypoparathyroidism, and serum calcium and iPTH levels after total thyroidectomy plus central neck dissection.

	PGPIEV score					
	0 (*n* = 27)	1 (*n* = 25)	2 (*n* = 56)	3 (*n* = 184)	4 (*n* = 316)	*P*
Age						0.298
≤45	14 (3.9)	13 (3.7)	39 (11.0)	101 (28.5)	188 (53.0)	
>45	13 (5.1)	12 (4.7)	17 (6.7)	83 (32.8)	128 (50.6)	
Sex ratio (M : F)	6 : 21	6 : 19	11 : 45	50 : 134	89 : 227	0.706
Hypocalcemia^†^ (*n* = 97)	21 (77.8)	15 (60.0)	15 (26.8)	18 (9.8)	31 (9.8)	<0.001
Hypoparathyroidism^‡^ (*n* = 12)	12 (44.4)	0 (0)	0 (0)	0 (0)	0 (0)	<0.001
Calcium on POD 1 (mg/dl)^*∗*^	2.01 (0.16)	2.07 (0.12)	2.10 (0.10)	2.16 (0.10)	2.17 (0.11)	<0.001^#^
iPTH on POD 1 (ng/l)^*∗*^	14.58 (4.53)	23.09 (5.09)	31.73 (6.59)	34.70 (9.32)	45.22 (14.91)	<0.001^§^
Proportion of iPTH on POD 1 of the initial value (before operation)^*∗*^	42.7 (16.7)	56.5 (11.5)	71.6 (17.7)	90.2 (23.2)	114.9 (27.5)	<0.001^§^

Values in parentheses are percentages unless indicated otherwise; ^*∗*^ values are mean (s.d.). ^†^Serum calcium concentration less than 8.0 mg/dl; ^‡^intact parathyroid hormone (iPTH) level below 15 ng/l; PGPIEV, parathyroid glands preserved *in situ* with excellent vascularity determined by the fine-needle pricking test. *χ*^2^ test, except ^#^one-way ANOVA, ^§^Kruskal–Wallis test.

## Data Availability

The data that support the findings of this study are available in the following link: https://figshare.com/s/5e96bb2c405d3dd1723a.

## Abbreviations

PTH: Parathyroid hormone
FNP: Fine-needle pricking
CND: Central neck dissection
PGPIEV: Parathyroid gland preserved *in situ* with excellent vascularity
iPTH: Intact parathyroid hormone
POD: Postoperative day.

## Supplementary Materials

Supplementary Materials Individual serum levels of intact parathyroid hormone (iPTH) during the perioperative period in different parathyroid glands preserved *in situ* with excellent vascularity (PGPIEV) groups (Figure S1 supplementary information). The mean serum levels of intact parathyroid hormone and calcium in the perioperative period depend on PGPIEV group classification (Table S1 supplementary information).
