# Supplementary material for: Fine-Needle Pricking Test of the Parathyroid Gland during Thyroid Surgery in Predicting Parathyroid Function
Source: Int J Endocrinol. 2022 Jun 25;2022:8747680. doi: 10.1155/2022/8747680 (PMC9252692; doi:10.1155/2022/8747680)
Supplement: Supplementary Materials — Individual serum levels of intact parathyroid hormone (iPTH) during the perioperative period in different parathyroid glands preserved in situ with excellent vascularity (PGPIEV) groups (Figure S1 supplementary information). The mean serum levels of intact parathyroid hormone and calcium in the perioperative period depend on PGPIEV group classification (Table S1 supplementary information). [file 8747680.f1.zip › 8747680.f1/Figure S1.docx]

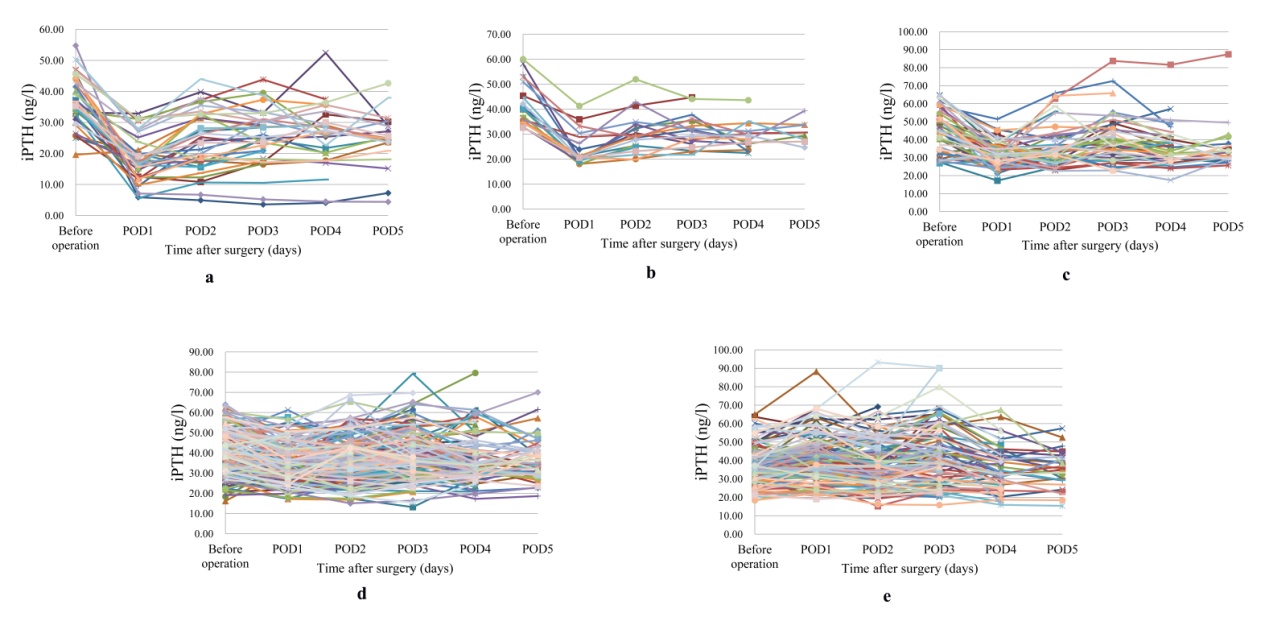


**Figure S1 Individual serum level of intact parathyroid hormone (iPTH) during the perioperative period, in different parathyroid gland preserved *in situ* with excellent vascularity (PGPIEV) groups.** a, PGPIEV group 0; b, PGPIEV group 1; c, PGPIEV group 2; d, PGPIEV group 3; e, PGPIEV group 4. POD, postoperative day.
