# Supplementary material for: Fine-Needle Pricking Test of the Parathyroid Gland during Thyroid Surgery in Predicting Parathyroid Function
Source: Int J Endocrinol. 2022 Jun 25;2022:8747680. doi: 10.1155/2022/8747680 (PMC9252692; doi:10.1155/2022/8747680)
Supplement: Supplementary Materials — Individual serum levels of intact parathyroid hormone (iPTH) during the perioperative period in different parathyroid glands preserved in situ with excellent vascularity (PGPIEV) groups (Figure S1 supplementary information). The mean serum levels of intact parathyroid hormone and calcium in the perioperative period depend on PGPIEV group classification (Table S1 supplementary information). [file 8747680.f1.zip › 8747680.f1/Table S1.docx]

**Table S1 Mean serum levels of intact parathyroid hormone and calcium in the perioperative period depend on PGPIEV group classification**

| PGPIEV  group | Biochemical values | Before operation | POD 1 | POD 2 | POD 3 | POD 4 | POD 5 |
| --- | --- | --- | --- | --- | --- | --- | --- |
| PGPIEV group 0 | iPTH (ng/l) | 35.90 (7.01) | 14.58 (4.53) | 19.48 (7.15) | 21.24 (7.65) | 21.87 (8.93) | 21.75 (7.60) |
|  | Calcium (mmol/l) | 2.38 (0.15) | 2.01 (0.16) | 1.94 (0.15) | 1.93 (0.17) | 1.99 (0.13) | 2.09 (0.14) |
| PGPIEV group 1 | iPTH (ng/l) | 41.59 (8.10) | 23.09 (5.09) | 29.83 (7.02) | 30.57 (5.82) | 27.74 (4.23) | 31.30 (4.78) |
|  | Calcium (mmol/l) | 2.35 (0.12) | 2.07 (0.12) | 2.01 (0.10) | 2.09 (0.09) | 2.15 (0.10) | 2.22 (0.08) |
| PGPIEV group 2 | iPTH (ng/l) | 45.93 (10.80) | 31.73 (6.59) | 35.69 (10.57) | 39.45 (12.20) | 35.78 (10.91) | 35.95 (12.68) |
|  | Calcium (mmol/l) | 2.40 (0.12) | 2.10 (0.10) | 2.09 (0.11) | 2.13 (0.12) | 2.17 (0.10) | 2.20 (0.11) |
| PGPIEV group 3 | iPTH (ng/l) | 39.63 (10.59) | 34.70 (9.32) | 36.47 (10.23) | 38.64 (11.19) | 36.79 (11.15) | 36.62 (9.90) |
|  | Calcium (mmol/l) | 2.40 (0.12) | 2.16 (0.10) | 2.15 (0.11) | 2.17 (0.13) | 2.22 (0.12) | 2.25 (0.11) |
| PGPIEV group 4 | iPTH (ng/l) | 40.00 (11.32) | 45.22 (14.91) | 42.21 (14.46) | 44.49 (15.56) | 39.20 (12.43) | 39.35 (13.14) |
|  | Calcium (mmol/l) | 2.41 (0.11) | 2.17 (0.11) | 2.16 (0.11) | 2.18 (0.11) | 2.25 (0.11) | 2.27 (0.11) |

Value is mean (s.d.). PGPIEV, parathyroid gland preserved *in situ* with excellent vascularity determined by fine-needle pricking test. PGPIEV group 0 (zero PGPIEV), PGPIEV group 1 (one PGPIEV), PGPIEV group 2 (two PGPIEV), PGPIEV group 3 (three PGPIEV), PGPIEV group 4 (four PGPIEV). iPTH, intact parathyroid hormone. POD, postoperative day.
